# Supplementary material for: Cellular senescence mediates fibrotic pulmonary disease
Source: Nat Commun. 2017 Feb 23;8:14532. doi: 10.1038/ncomms14532 (PMC5331226; doi:10.1038/ncomms14532)
Supplement: Supplementary Information — Supplementary Figures and Supplementary Tables. [file ncomms14532-s1.pdf]

**SUPPLEMENTARY INFORMATION**

**Supplementary Table 1.**

Demographic characteristics of IPF and control subjects analyzed by microarray.

| | **Control (n=64)** | **IPF, FVC ≥80% (n=17)** | **IPF, FVC 50-80% (n=60)** | **IPF, FVC <50% (n=16)** | |
|---|---|---|---|---|---|
| **Characteristic** | **Mean (±SD) or Number (%)** | | | | **p-value** |
| **Age** | 65.3 (10.2) | 65.2 (10.5) | 64.8 (7.96) | 59.2 (8.11) | 0.121[1] |
| **Sex** | | | | | 0.018[2] |
| **Male** | 34 (53.1%) | 11 (64.7%) | 48 (80%) | 11 (68.8%) | |
| **Female** | 30 (46.9%) | 6 (35.3%) | 12 (20%) | 5 (31.2%) | |
| **Height (inches)** | 66.4 (3.85) | 67.4 (3.28) | 68.5 (3.86) | 66.6 (3.78) | 0.021[1] |
| **Weight (pounds)** | 184 (44.6) | 193 (31.4) | 203 (37.6) | 198 (29.6) | 0.054[1] |
| **Pack-years** | 35.2 (33.1) | 33.6 (30.9) | 25.7 (20) | 25.9 (12.4) | 0.209[1] |
| **FVC Pre-Bronchodilator % predicted** | 93.6 (11.7) | 86.8 (5.71) | 65.8 (8.97) | 41.4 (6.9) | <0.001[1] |
| **Severity classification of DLCO** | 81.2 (17) | 58.2 (23) | 48.6 (12.9) | 26.6 (15.5) | <0.001[1] |
| **SF12 Physical Component Score** | 48.1 (11.5) | 35 (9.58) | 37.3 (10.9) | 28.6 (8.46) | <0.001[1] |
| **Meters walked in 6 minutes** | 419 (129) | 412 (64.1) | 385 (109) | 279 (128) | 0.002[1] |

[1]ANOVA F-test [2]Chi-Square

**Supplementary Table 2.**

Demographic characteristics of IPF and control subjects analyzed by RNA sequencing.

| | Control (n=19) | IPF (n=20) | |
|---|---|---|---|
| **Characteristic** | **Mean (±SD) or Number (%)** | | **p-value** |
| **Age** | 63.1 (14) | 57.2 (5.96) | 0.095[1] |
| **Sex** | | | 0.267[2] |
| **Male** | 9 (47.4%) | 14 (70%) | |
| **Female** | 10 (52.6%) | 6 (30%) | |
| **Height (inches)** | 65.6 (2.63) | 67.7 (3.1) | 0.031[1] |
| **Weight (pounds)** | 175 (30.4) | 178 (30.2) | 0.819[1] |
| **Pack-years** | 38.3 (37.8) | 27.2 (21.9) | 0.438[1] |
| **FVC Pre-Bronchodilator % predicted** | 96.2 (15.4) | 43 (12.3) | <0.001[1] |
| **Severity classification of DLCO** | 88.7 (16.7) | 25.3 (11.8) | <0.001[1] |
| **SF12 Physical Component Score** | 44 (9.04) | 26.1 (6.73) | <0.001[1] |
| **Meters walked in 6 minutes** | 373 (73.1) | 283 (79.9) | 0.006[1] |

[1]ANOVA F-test [2]Chi-Square

**Supplementary Table 3.**

Primer sequences used for real-time PCR.

| Gene | Species | Primer 1 | Primer 2 | Probe |
|---|---|---|---|---|
| **Col1a1** | Mouse | CATTGTGTATGCAGCTGACTTC | CGCAAAGAGTCTACATGTCTAGG | CCGGAGGTCCACAAAGCTGAACA |
| **Hprt** | Mouse | AGCAGGTCAGCAAAGAACT | CCTCATGGACTGATTATGGACA | ATTGTGGCCCTCTGTGTGCTCA |
| **Il6** | Mouse | TCCTTAGCCACTCCTTCTGT | AGCCAGAGTCCTTCAGAGA | CCTACCCCAATTTCCAATGCTCTCCT |
| **Ccl2 (Mcp1)** | Mouse | AACTACAGCTTCTTTGGGACA | CATCCACGTGTTGGCTCA | ACTCACCTGCTGCTACTCATTCACC |
| **Mmp10** | Mouse | TGTTGCTCTTCAGTATGTGTGT | CCAGGAATTGAGCCACAAGT | CGTCCTGGCATTGGGGTCAAACTC |
| **Mmp12** | Mouse | GCTCCTGCCTCACATCATAC | GGCTTCTCTGCATCTGTGAA | AGCTGTCTTTGACCCACTTCGCC |
| **Mmp3** | Mouse | TGTGGAGGACTTGTAGACTGG | GATGAACGATGGACAGAGGATG | TGGTTGCTGCTCATGAACTTGGC |
| **Cdkn2a (p16)** | Mouse | CTCTGCTCTTGGGATTGGC | GTGCGATATTTGCGTTCCG | TCCGCTGGGTGGTCTTTGTGTAC |
| **Serpine1 (Pai1)** | Mouse | CGTGTCAGCTCGTCTACAG | CTATGGTGAAACAGGTGGACT | ACCTTTGGTATGCCTTTCCACCCA |
| **Tbp** | Mouse | CCAGAACTGAAAATCAACGCAG | TGTATCTACCGTGAATCTTGGC | ACTTGACCTAAAGACCATTGCACTTCGT |
| **Tgfβ** | Mouse | CCGAATGTCTGACGTATTGAAGA | GCGGACTACTATGCTAAAGAGG | ATAGATGGCGTTGTTGCGGTCCA |
| **Tnf** | Mouse | TCTTTGAGATCCATGCCGTTG | AGACCCTCACACTCAGATCA | CCACGTCGTAGCAAACCACCAAGT |
| **Vcam1** | Mouse | TGTGCAGTTGACAGTGACA | GCAAAGGACACTGGAAAAGAG | TTCAGGGAATGAGTAGACCTCCACCT |
| **CCL2 (MCP1)** | Human | GCCTCTGCACTGAGATCTTC | AGCAGCCACCTTCATTCC | TCAATGCCCCAGTCACCTGCT |
| **CDKN1A (p21)** | Human | GAGACTAAGGCAGAAGATGTAGAG | GCAGACCAGCATGACAGAT | TTCCTCTTGGAGAAGATCAGCCGG |
| **CDKN2A (p16)** | Human | TGAGCTTTGGTTCTGCCATT | AGCTGTCGACTTCATGACAAG | TAGCAGTGTGACTCAAGAGAAGCCAGT |
| **IL6** | Human | CGGCTACATCTTTGGAATCTTC | GCCCAGCTATGAACTCCTTC | ACAAGCGCCTTCGGTCCAGTT |
| **TBP** | Human | TCGTGGCTCTCTTATCCTCAT | CAGTGAATCTTGGTTGTAAACTTGA | CGCAGCAAACCGCTTGGGATTAT |
| **ACTA2** | Human | GTGAAGAAGAGGACAGCACTG | CCCATTCCCACCATCACC | n/a |
| **COL1A1** | Human | AAGGGACACAGAGGTTTCAGTGG | CAGCACCAGTAGCACCATCATTTC | n/a |
| **COL1A2** | Human | CTTGCAGTAACCTTATGCCTAGCA | CCCATCTAACCTCTCTACCCAGTCT | n/a |
| **FN1** | Human | TGTCAGTCAAAGCAAGCCCG | TTAGGACGCTCATAAGTGTCACCC | n/a |
| **GAPDH** | Human | GGAAGGGCTCATGACCACAG | ACAGTCTTCTGGGTGGCAGTG | n/a |
| **STAT3** | Human | GAGATTGACCAGCAGTATAGCC | CAATCTCCATTGGCTTCTCAAG | n/a |

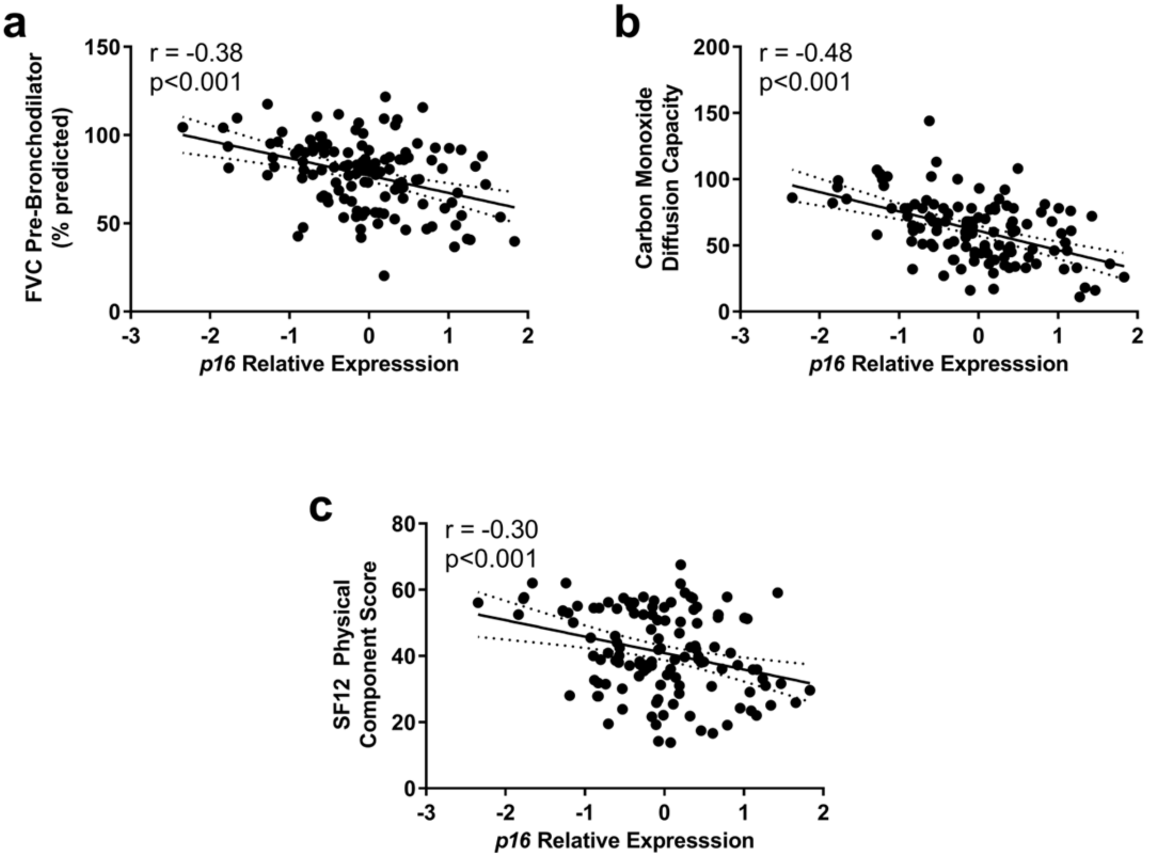


**Supplementary Figure 1.**

Increased p16 expression correlates with reduced pulmonary and physical function.

*p16* levels in lung specimens from control and IPF subjects were measured by microarray. Age-adjusted expression was compared to (a) forced vital capacity (FVC), (b) carbon monoxide diffusion capacity, and (c) SF12 PCS (control n=54, IPF n=72; Pearson correlation statistics are indicated).

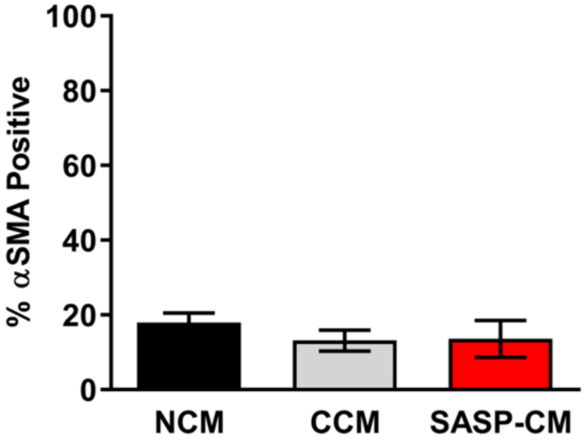


**Supplementary Figure 2.**

<u>Senescent epithelial cell SASP does not induce αSMA in fibroblasts.</u>

Normal bronchial epithelial cells were exposed to 5 Gy or sham conditions. Naïve IMR90 cells were treated with non-conditioned (NCM, black) or epithelial media collected 21-days post-irradiation (SASP-CM, red) or -sham exposure (gray). Following a 72 hour media treatment, the IMR90 cells were immunostained for αSMA and DAPI. Percentage αSMA positive cells were determined blindly, using a visual threshold (mean ± SEM; n=2 independent experiments).

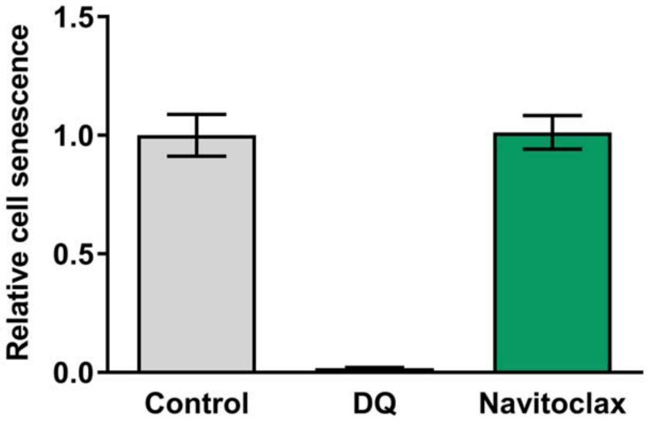


**Supplementary Figure 3.**

DQ reduces cellular senescence in etoposide-treated IMR90 fibroblasts.

Senescent IMR90 fibroblasts were treated for 48 hours with 20 μM D + 15 μM Q (yellow) or 10 μM navitoclax (green). Senescent cells were identified by staining for SA-β-gal activity, using the fluorogenic $C_{12}FDG$ substrate with an IN Cell 6000 microscope. Data are represented as the percentage of SA-β-gal positive cells normalized to control (untreated) cells (gray) (mean ± SEM; n=2).

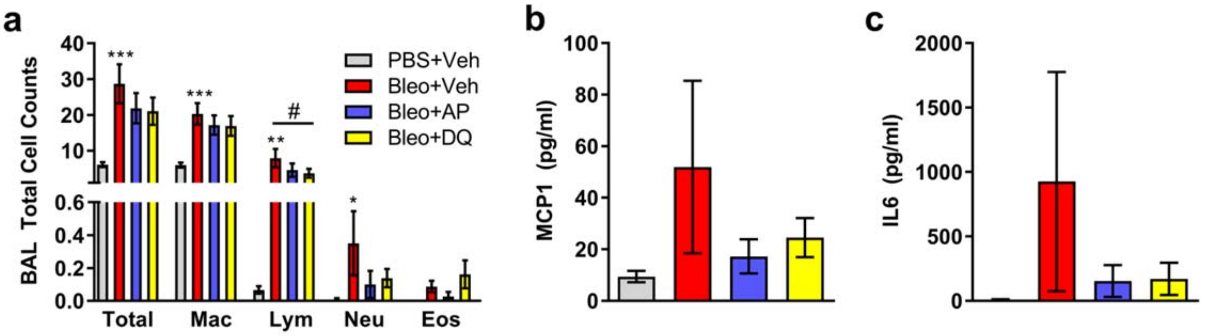


**Supplementary Figure 4.**

Bleomycin-induced inflammation is attenuated by senescent cell clearance.

(a) Total and differential inflammatory cells were counted, and immunoassays were used to profile (b) MCP1 and (c) IL6 within BAL fluid. (Mac=macrophages, Lym=lymphocytes, Neu=neutrophils, Eos=eosinophils; mean ± SEM; PBS+Veh n=13 (gray), Bleo+Veh n=8 (red), Bleo+AP n=12 (blue), Bleo+DQ n=13 (yellow); linear regression model; ***$p<0.0005$, **$p<0.005$, *$p\leq0.05$, ¥p=0.08, #p=0.1).

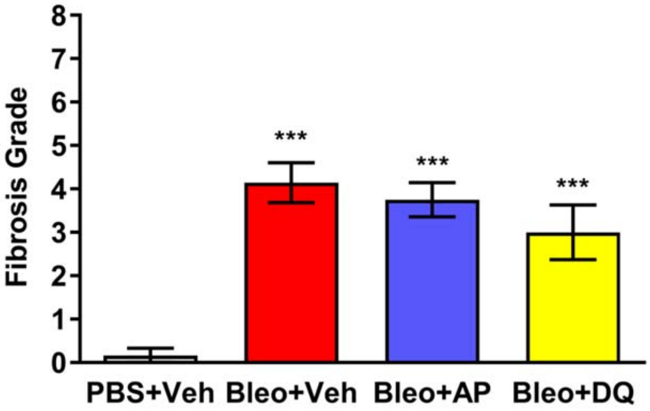


**Supplementary Figure 5.**

Fibrosis attenuation following senescent cell clearance is not significant.

H&E- and MT-stained FFPE lung sections corresponding to the indicated groups were scored by a blinded pathologist using a standardized, eight-tier Ashcroft scale.

(mean ± SEM; PBS+Veh n=12 (gray), Bleo+Veh n=7 (red), Bleo+AP n=12 (blue), Bleo+DQ=13 (yellow); ANOVA, ***$p<0.0005$).

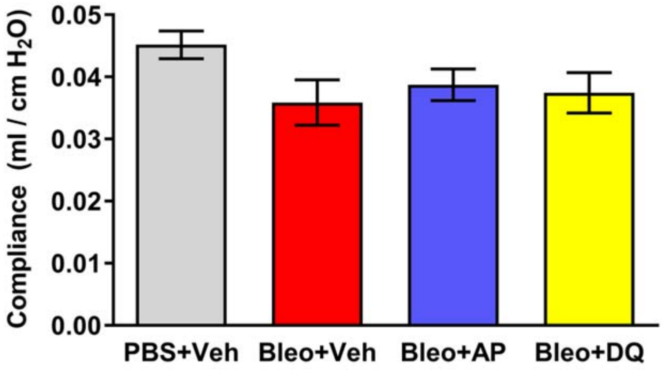


**Supplementary Figure 6.**

<u>Lung compliance following senescent cell clearance initiated in late-stage pathology.</u>

FlexiVent forced oscillation technique was used to assay lung compliance in Ink-Attac mice four weeks post- bleomycin or PBS exposure following two weeks of Veh, AP, or DQ treatment. (mean ± SEM; PBS+Veh n=6 (gray), Bleo+Veh n=13 (red), Bleo+AP n=15 (blue), Bleo+DQ=13 (yellow)).
